# Low temperature modulates natural peel degreening in lemon fruit independently of endogenous ethylene

**DOI:** 10.1093/jxb/eraa206

**Published:** 2020-05-06

**Authors:** Oscar W Mitalo, Takumi Otsuki, Rui Okada, Saeka Obitsu, Kanae Masuda, Yuko Hojo, Takakazu Matsuura, Izumi C Mori, Daigo Abe, William O Asiche, Takashi Akagi, Yasutaka Kubo, Koichiro Ushijima

**Affiliations:** 1 Graduate School of Environmental and Life Science, Okayama University, Okayama, Japan; 2 Institute of Plant Science and Resources, Okayama University, Kurashiki, Japan; 3 National Agriculture and Food Research Organization, Shikoku Research Station, Zentsuji, Japan; 4 Department of Research and Development, Del Monte Kenya Ltd, Thika, Kenya; 5 University of Trento, Italy

**Keywords:** 1-methylcyclopropene, carotenoids, chlorophyll, *Citrus limon*, ethylene, low temperature, peel degreening, phytohormones, transcriptome

## Abstract

Peel degreening is an important aspect of fruit ripening in many citrus fruit, and previous studies have shown that it can be advanced by ethylene treatment or by low-temperature storage. However, the important regulators and pathways involved in natural peel degreening remain largely unknown. To determine how natural peel degreening is regulated in lemon fruit (*Citrus limon*), we studied transcriptome and physiochemical changes in the flavedo in response to ethylene treatment and low temperatures. Treatment with ethylene induced rapid peel degreening, which was strongly inhibited by the ethylene antagonist, 1-methylcyclopropene (1-MCP). Compared with 25 ºC, moderately low storage temperatures of 5–20 °C also triggered peel degreening. Surprisingly, repeated 1-MCP treatments failed to inhibit the peel degreening induced by low temperature. Transcriptome analysis revealed that low temperature and ethylene independently regulated genes associated with chlorophyll degradation, carotenoid metabolism, photosystem proteins, phytohormone biosynthesis and signalling, and transcription factors. Peel degreening of fruit on trees occurred in association with drops in ambient temperature, and it coincided with the differential expression of low temperature-regulated genes. In contrast, genes that were uniquely regulated by ethylene showed no significant expression changes during on-tree peel degreening. Based on these findings, we hypothesize that low temperature plays a prominent role in regulating natural peel degreening independently of ethylene in citrus fruit.

## Teaser

Citrus peel degreening is promoted by low temperature via modulation of multiple genes associated with chlorophyll degradation, carotenoid biosynthesis, photosystem disassembly, phytohormones, and transcription factors without involving ethylene signalling.

## Introduction

Fruit ripening is a multifaceted process comprising various physiochemical and structural changes such as softening, degradation of starch to sugars, development of colour, and production of aroma volatiles (Cherian *et al.*, 2014; Seymour and Granell, 2014). In citrus fruit, colour development, commonly known as peel degreening, is a critical part of ripening and is characterized by a colour change in the peel from green to yellow/red/orange (Iglesias *et al.*, 2007). Peel degreening is an important aspect for marketability of citrus fruit (Porat, 2008), and hence there is wide interest in determining the fundamental regulatory mechanisms involved.

There are two main pathways that have been linked to citrus peel degreening. The first is chlorophyll degradation, which initially involves dephytilation of chlorophyll molecules by chlorophyllase (CLH) and pheophytinase (PPH), followed by removal of the central Mg atom by Mg-dechelatase to form pheophorbide. Pheophorbide is then converted to red chlorophyll catabolites (RCC) by pheophorbide a oxidase (PaO), and RCC is reduced to colourless compounds by RCC reductase (RCCR) (Hörtensteiner, 2006; Shimoda *et al.*, 2016; Yin *et al.*, 2016). The second pathway is carotenoid biosynthesis, which starts with the condensation of two geranylgeranyl pyrophosphate (GGPP) molecules by phytoene synthase (PSY) to form phytoene. Phytoene desaturase (PDS) and ζ-carotene desaturase (ZDS) successively convert phytoene to lycopene, which is then converted to α-carotene or β-carotene by lycopene ε-cyclase (LCYe) or lycopene β-cyclase (LCYb), respectively. α-carotene is later converted to lutein via sequential hydroxylation by ε-ring hydroxylase and β-ring hydroxylase (CHYb), whilst β-carotene is converted to zeaxanthin via β-cryptoxanthin by CHYb (Cunningham *et al.*, 1996; Ohmiya *et al.*, 2019). Genes encoding various enzymes for the main steps of chlorophyll degradation and carotenoid metabolism have been isolated and functionally characterized (Rodrigo *et al.*, 2013).

The phytohormone ethylene has been routinely used for commercial degreening in citrus fruit (Purvis and Barmore, 1981; Porat, 2008; Mayuoni *et al.*, 2011). Exogenous ethylene application has been shown to transcriptionally modulate both chlorophyll degradation and carotenoid metabolism (Rodrigo and Zacarias, 2007; Shemer *et al*., 2008; Yin *et al.*, 2016). Transcription factors (TFs) that may be involved in ethylene-induced peel degreening have also been identified and characterized (Yin *et al.*, 2016). Nevertheless, it remains unclear whether ethylene plays a role during natural degreening since citrus fruit are non-climacteric and the amounts of ethylene produced are minute (Eaks, 1970; Sawamura, 1981; Katz *et al.*, 2004).

Temperature has a large impact on a wide range of plant growth and developmental processes, including fruit ripening and maturation. Low temperature is thought to slow most cell metabolic activities, and hence it is the major postharvest technology used to delay fruit ripening and senescence (McGlasson *et al.*, 1979; Hardenburg *et al.*, 1986). However, promotion of fruit ripening by low temperature has been described in various fruit species, including kiwifruit (Kim *et al.*, 1999; Mworia *et al.*, 2012; Asiche *et al.*, 2017; Mitalo *et al.*, 2018*b*), European pears (El-Sharkawy *et al.*, 2003; Nham *et al.*, 2017), and apples (Tacken *et al.*, 2010). Recently, transcriptome studies have suggested that low temperature-specific genes might have regulatory roles during fruit ripening in kiwifruit (Asiche *et al.*, 2018; Mitalo et al., 2018*a*, 2019*a*, 2019*b*) and European pears (Mitalo *et al.*, 2019c).

Low temperature has also been linked to the promotion of fruit ripening (especially peel degreening) in several citrus fruit species. Typically, the seasonal temperature drops as most citrus fruit mature on the tree. Previous reports have correspondingly demonstrated that cold periods below 13 °C are required to stimulate on-tree fruit colour development (Manera et al., 2012, 2013; Rodrigo *et al.*, 2013; Conesa *et al.*, 2019). During storage, low/intermediate temperatures (6–15 °C) have also been shown to promote citrus peel degreening (Matsumoto *et al.*, 2009; Van Wyk *et al.*, 2009; Zhu *et al.*, 2011; Carmona *et al.*, 2012b; Tao *et al.*, 2012). Natural peel degreening in citrus fruit has to date been attributed to ethylene signalling, on the assumption that the basal System I ethylene levels produced by mature fruit are physiologically active (Goldschmidt *et al.*, 1993; Carmona *et al.*, 2012a). However, it remains unclear whether the colour changes in the peel during on-tree maturation and low-temperature storage are caused by basal ethylene, low temperature, and/or a synergistic effect of ethylene and low temperature, or because of another unknown mechanism.

In this study, we performed combined physiological and transcriptome analysis to examine the involvement of ethylene in low temperature-triggered degreening in citrus. Using lemon fruit, we found that peel degreening occurred faster at moderately low storage temperatures (5–20 °C; hereafter referred to simply as low temperature) compared to 25 °C. RNA-seq unveiled a distinct set of genes that were differentially regulated by low temperature independently of ethylene. Furthermore, repeated treatments of fruit with 1-methylcyclopropene (1-MCP), a well-known ethylene antagonist (Sisler and Serek, 1997; Watkins, 2006), failed to inhibit the low temperature-triggered degreening responses. Our results suggest that low temperature might modulate citrus peel degreening independently of basal endogenous ethylene.

## Materials and methods

### Plant material and treatments

Lemon fruit (*Citrus limon* L. cv. ‘Allen Eureka’) grown under standard cultural practices were collected in 2018 from a commercial orchard in Takamatsu, Kagawa, Japan. To examine on-tree degreening, sampling took place at seven time-points during fruit development on 3 Sept., 27 Sept., 12 Oct., 30 Oct., 14 Nov., 29 Nov., and 13 Dec., corresponding to 171, 196, 211, 230, 246, 261, and 276 d after full bloom (DAFB), respectively. To characterize postharvest effects of ethylene, fruit at 196 DAFB were divided into four groups containing 20 replicates each. The first group was used as the untreated control, the second group was treated with 2 µl l^–1^ 1-MCP for 12 h, the third group was continuously treated with 100 µl l^–1^ ethylene, while the fourth group was initially treated with 2 µl l^–1^ 1-MCP for 12 h followed by continuous treatment with 100 µl l^–1^ ethylene. 1-MCP was released by dissolving SmartFresh™ powder (AgroFresh, PA, USA) in water. All treatments were carried out at 25 °C for up to 8 d. For postharvest storage tests, fruit at 196 DAFB were divided into five groups of 40 replicates each, and stored at either 5, 10, 15, 20, or 25 °C for up to 42 d. In addition, three separate groups of 40 fruit each were stored at either 5, 15, or 25 °C with 1-MCP treatments for 12 h repeated twice a week. Samples of fruit peel (flavedo) were collected, frozen in liquid nitrogen, and stored at –80 °C for future analysis. At each sampling time, the flavedo was collected from each of three replicate fruits.

### Determination of the citrus colour index (CCI)

The *L*, *a*, and *b* Hunter lab parameters (Jiménez-Cuesta *et al.*, 1981) were measured at four even, equatorial sites on the fruit surface using a CR-200B chromameter (Konica Minolta). CCI values are presented as the results of 1000*a*/*Lb* transformation (Ríos *et al*., 2010), and are expressed as the mean value of five fruits.

### Determination of chlorophyll and carotenoid content

Chlorophylls were extracted and quantified from three replicate fruits per treatment according to the procedure described by Rodrigo *et al.* (2003), with slight modifications. Chlorophylls were extracted in 80% acetone and appropriate dilutions were used to quantify absorbance at 646.8 nm and 663.2 nm. The content was calculated from these measurements using the Lichtenthaler and Wellburn equations (Wellburn, 1994). Extraction and quantification of carotenoids were conducted using three replicate fruits per treatment according to the procedures described by Kato *et al.* (2004) and Matsumoto *et al.* (2007), with slight modifications. Briefly, carotenoids were successively extracted with 40% methanol and diethyl ether/methanol (containing 0.1% butylated hydroxytoluene). After saponification with methanolic potassium hydroxide, the organic layer of the extracts was vacuum-dried and analysed by HPLC. The HPLC analysis was carried out on an Extrema LC-4000 system (Jasco, Tokyo, Japan) equipped with a photodiode-array detector and autosampler. Samples were analysed on a Develosil C30-UG column (3 µm, 150×4.6 mm, Nomura Chemicals, Aichi, Japan) set at 20 °C and 0.5 ml min^–1^ flow rate. The UV-Vis spectra were obtained between 250 nm and 550 nm, and chromatograms were processed at 450 nm. The quantification of carotenoids was based on curves generated using authentic standards.

### Phytohormone measurements

Phytohormone extraction and analysis were performed according to the method described by Gupta *et al.* (2017), using deuterium-labelled internal standards for indole-3-acetic acid (IAA), abscisic acid (ABA), jasmonic acid (JA), gibberellins (GAs), *trans*-zeatin (tZ), N6-isopentenyladenine (iP), and salicylic acid (SA), and ^13^C-labelled jasmonoyl-*L*-isoleucine (JA-Ile). Eluted fractions were analysed on a 1260–6410 Triple Quad LC/MS system equipped with a ZOR-BAX Eclipse XDB-C18 column (Agilent Technologies). Liquid chromatography conditions are described in Supplementary Table S1 at *JXB* online, and the multiple-reaction-monitoring mode of the tandem quadrupole MS and precursor-product ion transitions for each compound are listed in Supplementary Table S2.

### RNA-seq and differential gene expression analysis

Total RNA was extracted from the flavedo of three replicate fruits from the control and ethylene groups after 4 d of treatment, as well as from fruit after 28 d of storage at 5, 15, or 25 °C. Illumina paired-end libraries were constructed using a NEBNext^®^ Ultra™ RNA Library Prep Kit for Illumina (New England Biolabs), before being sequenced using an Illumina HiSeq 2500 platform (Hokkaido System Co. Ltd., Japan). Trimming was done to obtain ≥10 million paired reads per sample, and the reads were mapped to the reference *Citrus clementina* Genome v1.0 (Wu *et al.*, 2014). Gene expression levels were calculated using the reads per kilobase per million (RPKM) method and differentially expressed genes (DEGs) were identified using false-discovery rates (FDR) analysis (Robinson *et al.*, 2010). DEG selection was based on two criteria, namely genes with RPKM≥3.0 and FDR≤0.001, and fold-change≥3.0 in the mean RPKM for ethylene versus control, 5 °C versus 25 °C, and/or 15 °C versus 25 °C. For co-expression analysis, the weighted gene co-expression network analysis (WGCNA) method (Zhang and Horvath, 2005) was used to generate modules of highly correlated genes based on the RNA-seq expression data. Gene modules were identified by implementing the WGCNA package in R (Langfelder and Horvath, 2008). The soft-thresholding power and tree-cut parameters used for the WGCNA analysis were 12 and 0.15, respectively. Significantly enriched gene ontology (GO) terms were established using the agriGO (v.2.0) web-based toolkit (Tian *et al.*, 2017), using hypergeometric tests followed by a Bonferroni correction to calculate a *P*-value for each term. We used *P*<0.05 as the cut-off for a significantly enriched GO term.

### Reverse-transcriptase quantitative PCR (RT-qPCR)

Total RNA was extracted from the flavedo of fruit at the time of sampling from the tree (0 d, on-tree fruit), after 4 d for the ethylene, 1-MCP+ethylene, and control groups, and after 28 d storage at either 5, 10, 15, 20, or 25 °C. To remove genomic DNA contamination from the extracted RNA, treatment with DNase I (Nippon Gene, Tokyo, Japan) was carried out, followed by clean-up with FavorPrep after using a Tri-Reagent RNA Clean-up Kit (Favorgen Biotech. Co., Ping-Tung, Taiwan). For all treatments, 2.4 µg of clean RNA was reverse-transcribed to cDNA using a TaKaRa RNA PCR™ kit. Gene-specific primers were designed using the Primer3 software (v.0.4.0, http://bioinfo.ut.ee/primer3-0.4.0/;Supplementary Table S3). The gene expression of three biological replicates was examined using a MYiQ Single-Color Reverse Transcriptase-Quantitative PCR Detection System (Bio-Rad) using TB Green™ Premix ExTaq™ II (Takara). *AcActin* (*Ciclev10025866m.g*) was used as the housekeeping gene after examining its constitutive expression pattern in the RNA-seq results. Relative expression values were calculated using fruit at 196 DAFB (0 d).

### Statistical analysis

Data were subjected to statistical analysis using R v.3.4.0 (www.r-project.org). Differences in CCI, pigment and phytohormone contents, and gene expression were determined using ANOVA followed by Tukey’s *post hoc* test.

## Results

### Ethylene-induced degreening

**Fig. 1.**
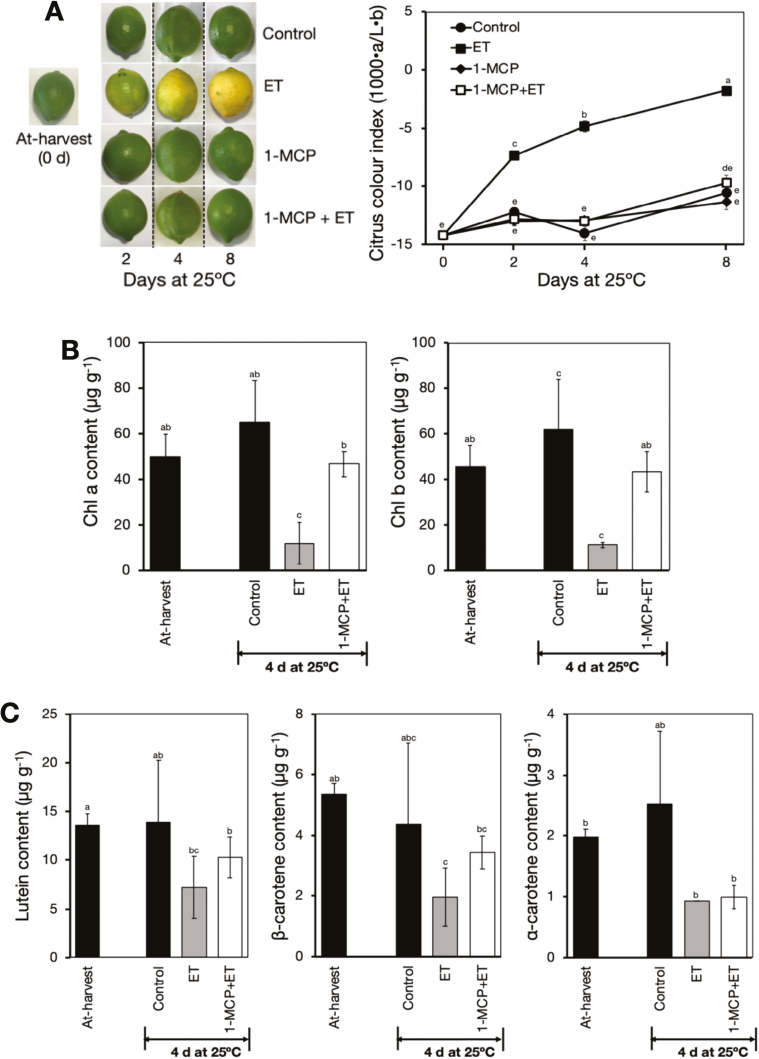
Ethylene-induced peel degreening in detached lemon fruit. (A) Appearance and colour index of fruit in response to treatment with ethylene and/or 1-methylcyclopropene (1-MCP). Fruit were either untreated (control), continuously treated with 100 µl l^–1^ ethylene (ET), treated with 2 µl l^–1^ 1-MCP for 12 h (1-MCP), or pre-treated with 2 µl l^–1^ 1-MCP for 12 h followed by continuous treatment with 100 µl l^–1^ ethylene (1-MCP+ET). An increase in the colour index indicates the fruit changing from green to yellow. (B) Changes in peel chlorophyll contents in response to ethylene and/or 1-MCP treatments. (C) Changes in peel carotenoid contents in response to ethylene and/or 1-MCP treatments. Data are means (±SE) of five replicate fruits. Different letters indicate significant differences between means as determined using ANOVA followed by Tukey’s test (*P*<0.05).

In response to ethylene treatment, a colour change was initiated in the peel from green to yellow after 2 d, and a full yellow colour developed after 8 d (Fig. 1A). This colour change was indicated by a rapid increase in CCI from –14.2 at harvest (0 d) to –1.8 after 8 d. However, it was notable that fruit pre-treated with 1-MCP followed by continuous ethylene treatment retained their greenish colour and showed no significant changes in CCI throughout the experimental period.

To further determine the role of ethylene in citrus peel degreening, we examined changes in the major colour pigments, namely chlorophylls and carotenoids. Both chlorophyll *a* and *b* in the peel decreased drastically upon ethylene treatment (from ~50 µg g^–1^ at 0 d to only 11 µg g^–1^ at 4 d), and pre-treatment with 1-MCP completely abolished this effect (Fig. 1B). Lutein, β-carotene, and α-carotene were the major carotenoids in the peel of the fruit (Supplementary Fig. S1), and these results were in close agreement with those of Agócs *et al.* (2007). Interestingly, all the three identified carotenoids displayed a substantial decrease after 4 d of ethylene treatment, and the ethylene-induced reduction was also inhibited by pre-treatment with 1-MCP (Fig. 1C). Taken together, these results demonstrated that ethylene played a key role in regulating peel degreening, and that 1-MCP treatment effectively rendered the fruit insensitive to ethylene.

### Peel degreening at different storage temperatures and the effects of 1-MCP

**Fig. 2.**
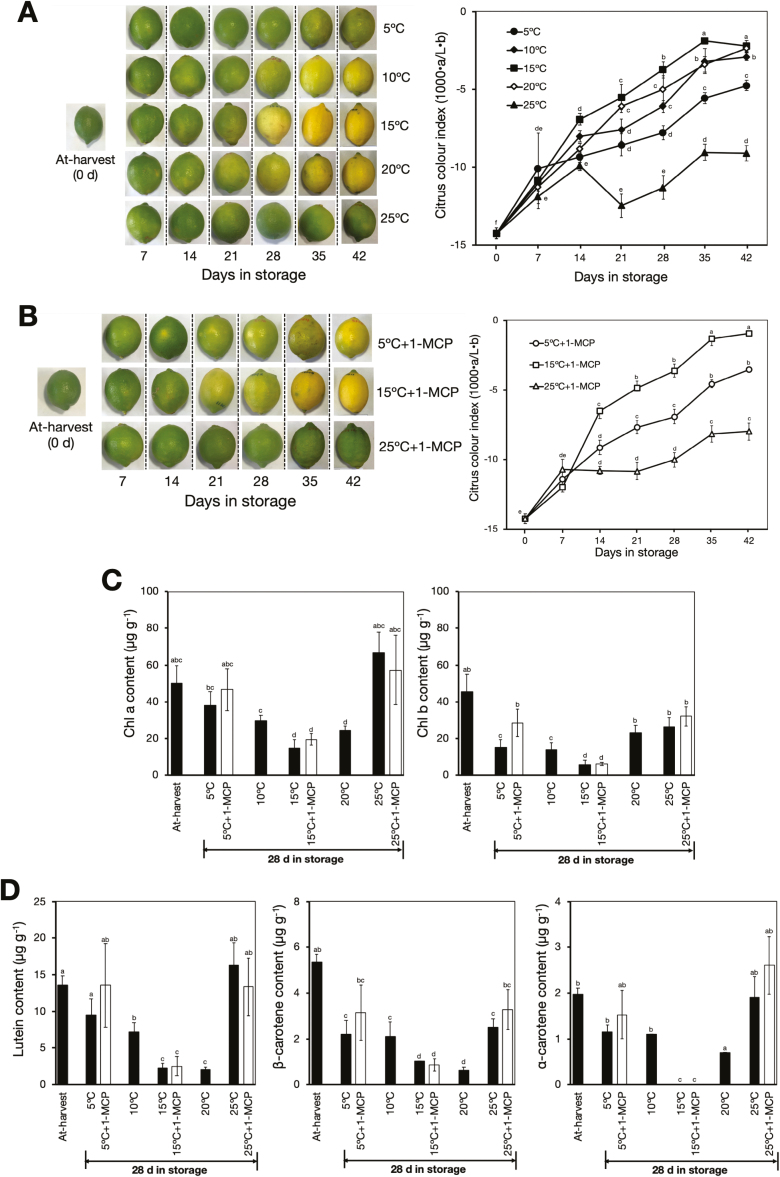
Promotion of peel degreening by low temperature in detached lemon fruit. (A) Appearance and colour index of fruit during storage at 5–25 °C in an ethylene-free environment. An increase in the colour index indicates the fruit changing from green to yellow. (B) Effects of 1-MCP treatment on peel colour changes during storage at 5, 15, or 25 °C. Treatments with 1-MCP (2 µl l^–1^) were carried out twice a week to keep fruit insensitive to ethylene. (C) Peel chlorophyll contents at different storage temperatures with or without 1-MCP treatment. (D) Peel carotenoid contents during storage at different temperatures with or without 1-MCP treatment. Data are means (±SE) of five replicate fruits. Different letters indicate significant differences between means as determined using ANOVA followed by Tukey’s test (*P*<0.05)

Changes in peel colour in detached fruit were also investigated during storage at different temperatures. The colour of the fruit at 5–20 °C gradually changed from green to yellow, with a concomitant increase in CCI to about –2.3 after 28–42 d (Fig. 2A). Peel degreening was most pronounced at 15 °C, followed by 10 °C and 20 °C, whereas at 5 °C the fruit retained an appreciable greenish colour even after 42 d. In contrast, fruit at 25 °C retained their greenish colour and there were minimal changes in CCI across the storage period. These results indicated that moderately low storage temperatures promoted peel degreening in lemon fruit.

To determine whether the basal levels of System I ethylene present in the mature fruit played a role in the degreening that we observed at low temperature, we treated fruit at 5, 15, and 25 °C twice a week with 1-MCP. Surprisingly, comparable degreening was observed in fruit at 5 °C and 15 °C but not at 25 °C, notwithstanding the repeated 1-MCP treatments (Fig. 2B). This suggested that low temperature might have promoted the colour changes in the fruit peel independently of ethylene.

The promotion of degreening by low temperature was further investigated through determination of the chlorophyll and carotenoid contents of the peel. We found that there were substantial decreases in chlorophyll *a* and *b* contents after 28 d of storage at moderately low temperatures (5–20 °C), whereas no significant changes were observed at 25 °C (Fig. 2C). The chlorophyll content also decreased in fruit at 5 °C and 15 °C despite repeated 1-MCP treatments. Similar to the ethylene experiment, we observed a substantial decrease in the peel contents of lutein, β-carotene, and α-carotene at 5–20 °C, while there were no observable changes at 25 °C (Fig. 2D). However, it was notable that repeated 1-MCP treatments did not inhibit the reduction in the contents of the carotenoids at 5 °C and 15 °C. Taken together, these results indicated that, relative to 25 °C, low storage temperatures modulated peel degreening in lemon fruit, possibly in an ethylene-independent manner.

### Differential expression analysis in the flavedo

#### Overview of the changes in the transcriptome.

To gain deeper insights into the mechanisms of low-temperature promotion of peel degreening, we conducted a comprehensive transcriptome analysis to compare the temperature-induced responses with those activated by ethylene. The ethylene-induced responses were examined by comparing flavedo samples from controls with those of fruit subjected to 4 d of ethylene treatment. For the responses triggered by low temperature, samples obtained after 28 d of storage at either 5 °C or 15 °C were compared with those at 25 °C.

**Fig. 3.**
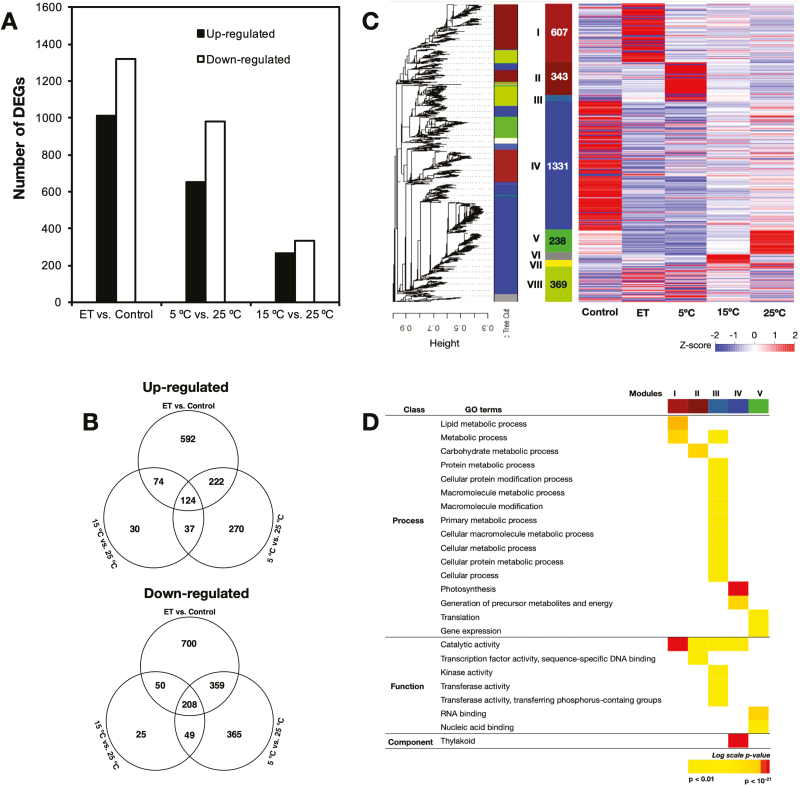
Global transcriptome changes induced by ethylene treatment and low temperature treatments in the flavedo of detached lemon fruit. (A) The numbers of differentially expressed genes (DEGs) in response to ethylene (ET), 5 °C, and 15 °C. (B) Venn diagrams showing the up- and down-regulated DEGs in response to ethylene, 5 °C, and 15 °C. (C) Weighted gene co-expression network analysis (WGCNA) and subsequent heatmap visualization of the 3105 DEGs from RNA-seq data. Each end branch in the tree represents one of the DEGs and each major branch represents one of the modules. The panel with total gene numbers shows the eight different modules obtained after implementing the tree cut-off (0.15) to merge close modules. The panel at the right shows a heatmap visualization of the expression measures of DEGs in each module in response to ethylene and the different storage temperatures. (D) Selected GO terms enriched among the WGCNA co-expressed DEGs in response to ethylene, 5 °C, and 15 °C.

RNA-seq analysis identified 3105 DEGs (*q*-value<0.001) that were regulated by either ethylene or low temperature (Fig. 3). Of these, ethylene accounted for 2329, compared with the 5 °C and 15 °C treatments that accounted for 1634 and 597, respectively (Fig. 3A). In all treatments, the number of down-regulated DEGs was higher than that of up-regulated ones. Ethylene treatment exclusively up- and down-regulated 592 and 700 genes, respectively (Fig. 3B). A combined total of 337 and 439 genes were exclusively up- and down-regulated, respectively, by the 5 °C and 15 °C treatments. The remaining DEGs (420 up-regulated and 617 down-regulated) were jointly influenced by ethylene, 5 °C and/or 15 °C. Subsequent WGCNA of the total 3105 DEGs identified eight modules (clusters of highly interconnected genes; Fig. 3C), and heatmapping indicated that the largest of these modules comprised genes that were down-regulated by ethylene or low temperature. The second largest was the module comprising genes exclusively up-regulated by ethylene. The remaining modules included genes that were differentially regulated by both ethylene and low temperature.

Based on the WGCNA results, we performed GO classification to further examine the molecular changes involved. Dominant GO terms in Module I (comprising genes specifically up-regulated by ethylene) were ‘metabolic process’ and catalytic activity’ (Fig. 3D). There was also significant enrichment of DEGs associated with ‘metabolic process’, ‘macromolecule modification’, ‘cellular process’, ‘photosynthesis’, ‘thylakoid’, ‘metabolite precursors’, ‘translation’, ‘gene expression’ and ‘catalytic’ activity in Modules II, III, IV, and V, indicating that they were enriched by both ethylene and low temperature. Other GO terms that were enriched in response to ethylene or low temperature were related to ‘transcription factor activity’, ‘transferase activity’, ‘RNA binding’, and ‘nucleic acid binding’. No enriched GO terms were detected for DEGs in the Modules VI, VII, and VIII. It was notable that significant enrichment of DEGs related to ‘photosynthesis’ and ‘catalytic’ activity was observed in response to ethylene, 5 °C, and 15 °C (Supplementary Fig. S2). Overall, the DEGs identified could be pooled into three distinct groups. The first group comprised ethylene-specific genes, the second group included low temperature-specific genes, and the third group consisted of genes regulated by either ethylene or low temperature. Detailed information about the DEGs showing specific and shared responses to ethylene, 5 °C, and/or 15 °C is given in Supplementary Tables S4–S10.

#### Transcripts associated with chlorophyll metabolism.

**Fig. 4.**
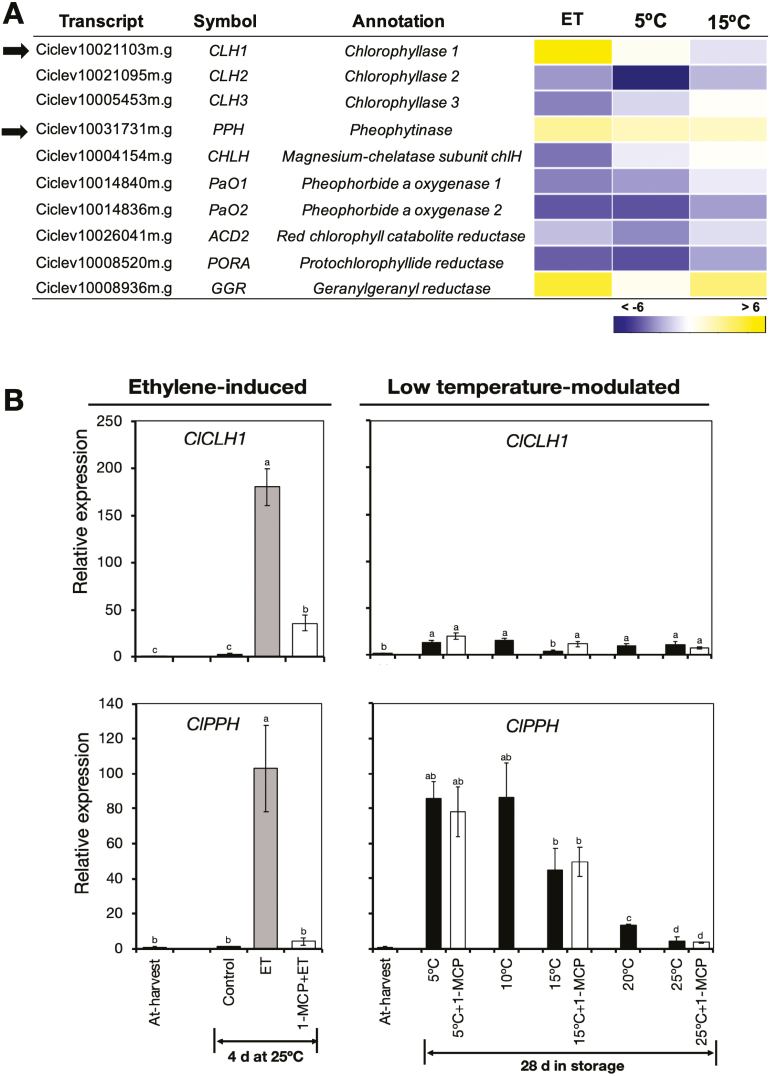
Expression of genes related to chlorophyll metabolism in the flavedo of detached lemon fruit in response to ethylene or low temperatures. (A) Heatmap showing identified differentially expressed genes associated with chlorophyll metabolism in fruit exposed to ethylene, 5 °C, or 15 °C. The data are expressed as the log_2_ value of fold-change for ethylene (ET) versus control, 5 °C versus 25 °C, and 15 °C versus 25 °C. (B) RT-qPCR analysis of the expression of *chlorophyllase 1* (*ClCLH1*) and *pheophytinase* (*ClPPH*), indicated by the arrows in (A), which are known to be involved in chlorophyll degradation in many plant species. Expression is relative to the value at harvest and the housekeeping gene was *AcActin*. Data are means (±SE) of three replicate fruits. Different letters indicate significant differences between means as determined using ANOVA followed by Tukey’s test (*P*<0.05).

One of the main processes contributing to peel degreening is chlorophyll degradation (Hörtensteiner, 2006). We therefore examined the expression patterns of chlorophyll metabolism genes in the RNA-seq data, and found that only three of the 10 identified DEGs were up-regulated by ethylene or low temperature (Fig. 4A, Supplementary Table S11). Of these genes, two (*ClCLH1* and *ClPPH*) have been previously associated with chlorophyll degradation in citrus fruit (Jacob-Wilk *et al.*, 1999; Yin *et al.*, 2016). RT-qPCR analysis confirmed that *ClCLH1* was up-regulated only in response to ethylene treatment, while expression of *ClPPH* increased in response to ethylene or low temperature (Fig. 4B). Interestingly, however, repeated 1-MCP treatments failed to suppress the increased expression levels of *ClPPH* at 5 °C and 15 °C despite a single treatment inhibiting ethylene-induced changes.

#### Transcripts associated with carotenoid metabolism.

**Fig. 5.**
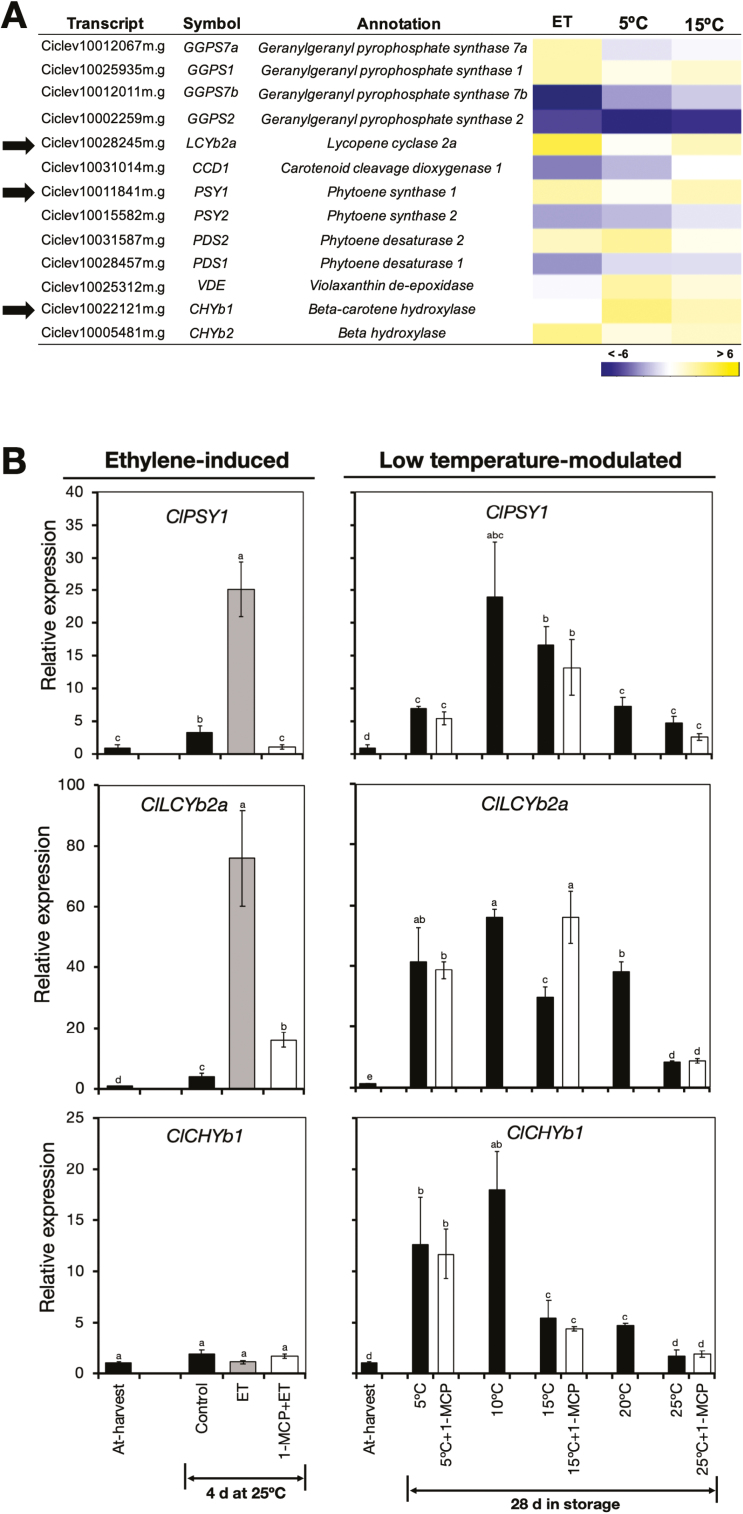
Expression of genes related to carotenoid metabolism in the flavedo of detached lemon fruit in response to ethylene or low temperatures. (A) Heatmap of identified differentially expressed genes associated with carotenoid metabolism in fruit exposed to ethylene, 5 °C, or 15 °C. The data are expressed as the log_2_ value of fold-change for ethylene (ET) versus control, 5 °C versus 25 °C, and 15 °C versus 25 °C. (B) RT-qPCR analysis of the expression of *phytoene synthase 1* (*ClPSY1*), *lycopene cyclase 2a* (*ClLCYb2a*), and *β-carotene hydroxylase 1* (*ClCHYb1*), indicated by the arrows in (A). Expression is relative to the value at harvest and the housekeeping gene was *AcActin*. Data are means (±SE) of three replicate fruits. Different letters indicate significant differences between means as determined using ANOVA followed by Tukey’s test (*P*<0.05).

The other process that leads to peel degreening is carotenoid metabolism, and the RNA-seq data identified 13 DEGs associated with this (Fig. 5A, Supplementary Table S11). Of these genes, *ClPSY1*, *ClLCYb2a*, and *ClCHYb1* displayed high RPKM values and unique expression patterns, and we selected them for further analysis by RT-qPCR. This revealed that *ClPSY1* and *ClLCYb2a* were up-regulated during both ethylene-induced and low temperature-modulated degreening, while *ClCHYb1* was up-regulated exclusively by low temperature (Fig. 5B). The expression of all three genes increased in the peel of fruit at 5 °C and 15 °C despite repeated 1-MCP treatments.

#### Transcripts encoding photosystem proteins.

**Fig. 6.**
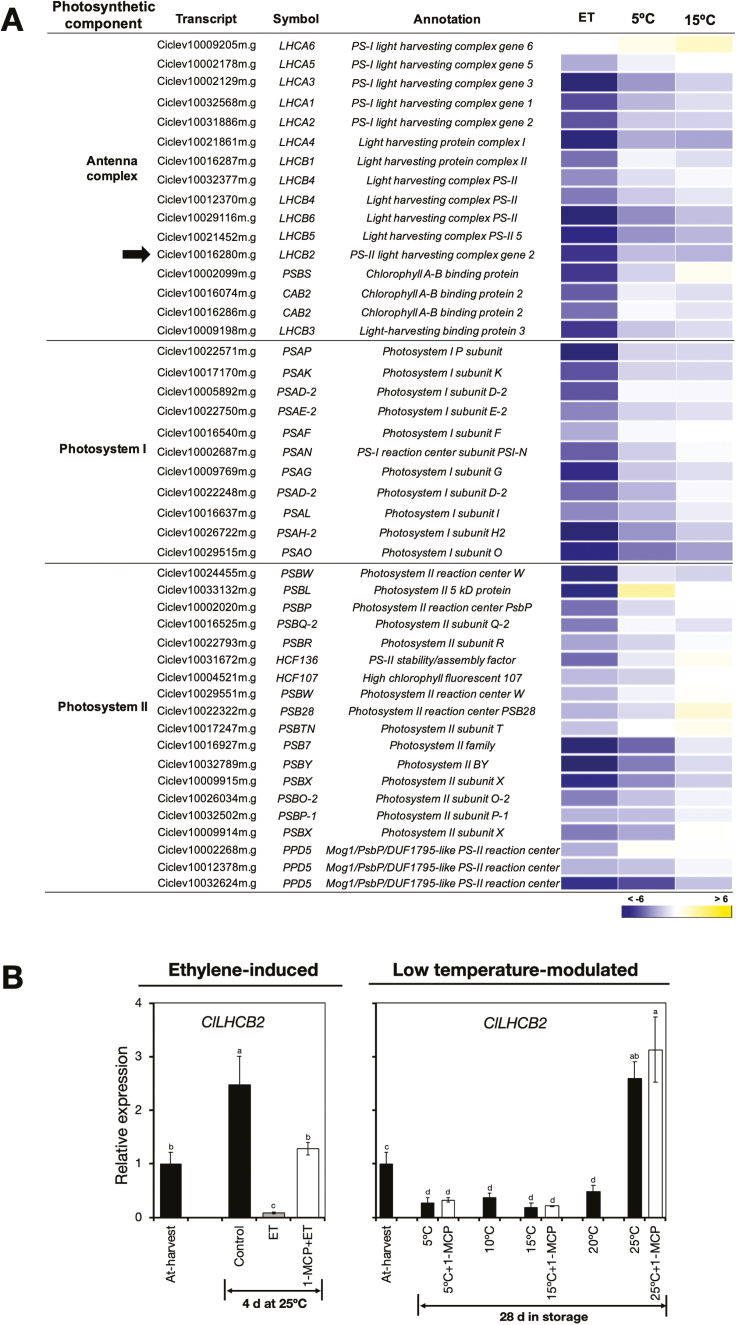
Changes in the expression of genes encoding photosystem proteins in the flavedo of detached lemon fruit in response to ethylene or low temperatures. (A) Heatmap of identified differentially expressed genes encoding photosystem proteins in fruit exposed to ethylene, 5 °C, or 15 °C. The data are expressed as the log_2_ value of fold-change for ethylene (ET) versus control, 5 °C versus 25 °C, and 15 °C versus 25 °C. (B) RT-qPCR analysis of the expression of *light harvesting complex 2* (*ClLHCB2*), indicated by the arrow in (A). Expression is relative to the value at harvest and the housekeeping gene was *AcActin*. Data are means (±SE) of three replicate fruits. Different letters indicate significant differences between means as determined using ANOVA followed by Tukey’s test (*P*<0.05).

Genes encoding photosystem proteins also featured prominently among the identified DEGs, and most of them were down-regulated by both ethylene treatment and low temperature (Fig. 6A, Supplementary Table S11); however, ethylene appeared to have the greater influence on their down-regulation. Since most of the genes in this category showed a similar expression pattern, we selected only one, *light harvesting complex 2* (*ClLHCB2*), for validation and further analysis by RT-qPCR, and the results confirmed that both ethylene treatment and low temperature caused a reduction in expression levels (Fig. 6B). Nevertheless, repeated 1-MCP treatments did not suppress the decrease in expression induced at 5 °C and 15 °C, suggesting that the influence of low temperature on *ClLHCB2* expression was independent of ethylene.

#### Phytohormone levels and associated transcripts.

**Fig. 7.**
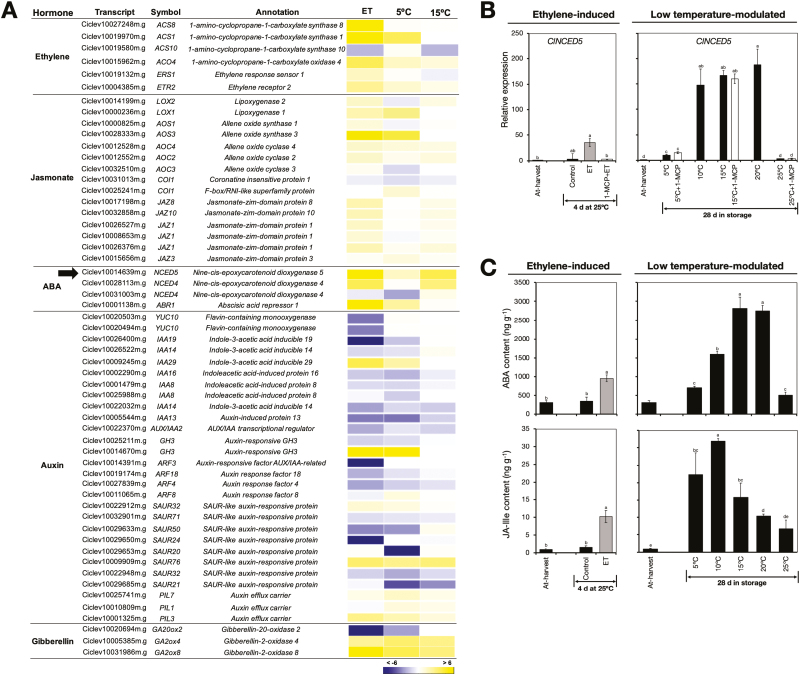
Levels of phytohormones and the expression of associated genes in the flavedo of detached lemon fruit. (A) Heatmap showing differentially expressed genes encoding proteins associated with phytohormone biosynthesis and signalling in fruit exposed to ethylene or low temperatures. The data are expressed as the log_2_ value of fold-change for ethylene (ET) versus control, 5 °C versus 25 °C, and 15 °C versus 25 °C. (B) RT-qPCR analysis of the ABA biosynthetic gene *9-cis-epoxycarotenoid dioxygenase 5* (*ClNCED5*), indicated by the arrow in (A). Expression is relative to the value at harvest and the housekeeping gene was *AcActin*. (C) Levels of ABA and JA-Ile in lemon fruit treated with ethylene or after 28 d storage at the indicated temperatures. Data are means (±SE) of three replicate fruits. Different letters indicate significant differences between means as determined using ANOVA followed by Tukey’s test (*P*<0.05).

Another prominent category among the identified DEGs included genes that were associated with the biosynthesis and signalling of phytohormones, especially ethylene, JA, ABA, auxin, and GA (Fig. 7A). Most of the ethylene-related genes were up-regulated by ethylene treatment, while low temperature only showed a slight effect on their expression, especially at 5 °C. Genes that were associated with JA and ABA were mostly up-regulated by both ethylene treatment and low temperature. Auxin-related genes showed varied expression patterns, although the general trend was towards down-regulation by both ethylene treatment and low temperature. We also identified three GA-associated DEGs, of which one, *ClGA20ox2* that is associated with GA biosynthesis, was down-regulated by both ethylene treatment and low temperature, especially at 5 °C. In contrast, *ClGA2ox4* and *ClGA2ox8*, which are associated with GA degradation, were up-regulated by ethylene treatment and by low temperature. To further examine the roles of ethylene and low temperature in the regulation of phytohormone-related genes, we selected *9-cis-epoxycarotenoid dioxygenase* (*ClNCED5*), which is associated with ABA biosynthesis, for additional analysis by RT-qPCR. The results confirmed that it was up-regulated both after 4 d of ethylene exposure and 28 d of storage at low temperatures (5–20 °C; Fig. 7B). There was also a significant increase in *ClNCED5* expression in fruit that were repeatedly treated with 1-MCP at 5 °C and 15 °C. The transcript levels of *ClNCED5* were notably higher in fruit stored at low temperature than in fruit treated with ethylene.

The results described above motivated us to determine the phytohormone content in the flavedo of fruit exposed to ethylene and different storage temperatures. The results indicated that both caused a significant increase in ABA and JA-Ile levels (Fig. 7C) In particular, levels were substantially higher in fruit stored at low temperatures than in fruit treated with ethylene. Unfortunately, we were not able to detect other hormones because of their extremely low endogenous levels and severe ion suppression effects during the LC/MS analysis.

#### Transcripts encoding transcription factors.

**Fig. 8.**
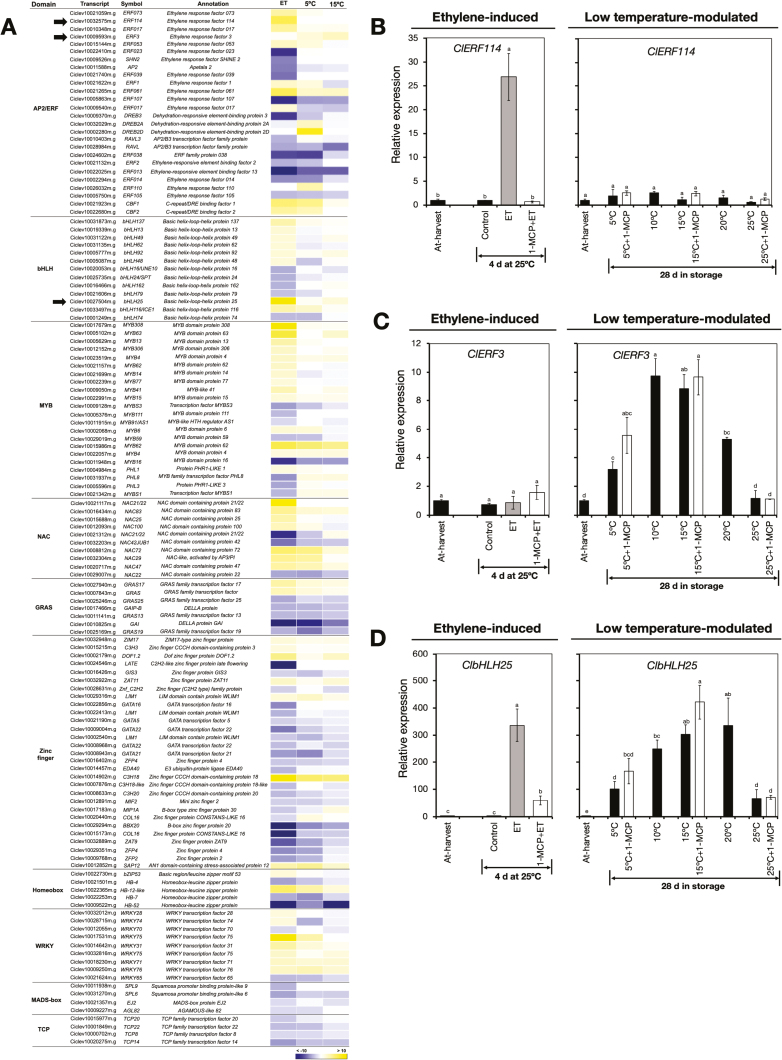
Changes in expression of genes encoding transcription factors in the flavedo of detached lemon fruit. (A) Heatmap showing identified differentially expressed genes encoding various transcription factors in fruit exposed to ethylene or low temperatures. The data are expressed as the log_2_ value of fold-change for ethylene (ET) versus control, 5 °C versus 25 °C, and 15 °C versus 25 °C. (B–D) RT-qPCR analysis of the relative expression of (B) *ClERF114*, (C) *ClERF3*, and (D) *ClbHLH25*, as indicated by the arrows in (A). Expression is relative to the value at harvest and the housekeeping gene was *AcActin*. Data are means (±SE) of three replicate fruits. Different letters indicate significant differences between means as determined using ANOVA followed by Tukey’s test (*P*<0.05).

A total of 128 DEGs in the RNA-seq data were found to encode a wide range of putative TF families, including AP2/ERF, bHLH, MYB, NAC, GRAS, zinc finger, homeobox, WRKY, MADS, and TCP (Fig. 8A, Supplementary Table S11). This underscored the relevance of TF activity in the peel degreening process of lemon fruit. We pooled the identified genes into three distinct groups, namely those that were influenced by ethylene only, such as *ClERF114*, those that were influenced by low temperature only, such as *ClERF3*, and those that were influenced by both ethylene and low temperature, such as *ClbHLH25*. RT-qPCR analysis confirmed that *ClERF114* was exclusively up-regulated by ethylene treatment as its expression was maintained at minimal levels during storage (Fig. 8B). In contrast, *ClERF3* was exclusively up-regulated by low temperature since marginal expression levels were observed in ethylene-treated fruit (Fig. 8C). Finally, *ClbHLH25* expression increased both upon ethylene treatment and after storage at temperatures lower than 25 °C (Fig. 8D). It was also notable that repeated 1-MCP treatments failed to abolish the up-regulation of *ClERF3* and *ClbHLH25* at 5 °C and 15 °C (Fig. 8C, D).

### On-tree peel degreening and expression analysis of associated genes

**Fig. 9.**
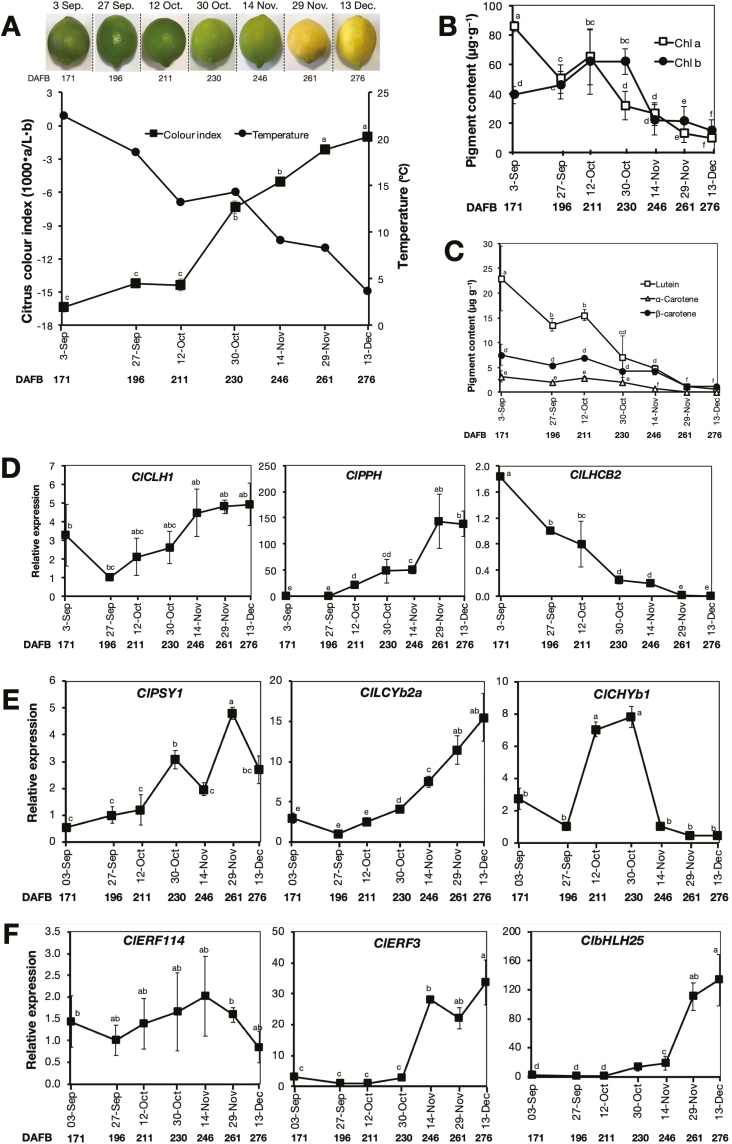
Changes in peel colour and gene expression analysis in lemon fruit during on-tree maturation. (A) Appearance and citrus colour index of representative fruit at different developmental stages together with data for minimum ambient temperatures. An increase in the colour index indicates the fruit changing from green to yellow. The temperature data were accessed from the website of Japan Meteorological Agency (http://www.data.jma.go.jp/obd/stats/etrn/view/daily_s1.php?prec_no=72&block_no=47891&year=2014&month=12&day=&view=p1). (B) Chlorophyll *a* and *b* contents at different developmental stages. (C) Levels of lutein, α-carotene, and β-carotene at different developmental stages. (D–F) RT-qPCR analysis of the expression of selected genes associated with chlorophyll metabolism and photosystem proteins (D), carotenoid metabolism (E), and transcription factors (F) at different developmental stages. Expression is relative to the value at harvest and the housekeeping gene was *AcActin*. Data are means (±SE) of five replicate fruits. Different letters indicate significant differences between means as determined using ANOVA followed by Tukey’s test (*P*<0.05).

The roles of ethylene and low temperature in natural peel degreening were investigated during on-tree maturation of lemon fruit. Fruit were harvested at seven progressive stages ranging from 171–276 DAFB between early September and mid-December. Peel colour progressively changed from green on 3 September to full yellow on 13 December, which was indicated by a concomitant increase in CCI from –16.3 to –1.1 (Fig. 9A). During this period, the mean minimum temperature in the orchard decreased gradually from 22.5 °C to 3.7 °C. The increase in CCI was initially slow between 3 September to 12 October (from –16.3 to –14.2) when the minimum temperatures were above 13 °C; however, CCI increased rapidly between 12 October and 13 December (from –14.2 to –1.1) when the minimum temperatures were below 13 °C. The observed loss of green colour during on-tree maturation corresponded closely with a gradual decrease in the chlorophyll *a* and *b* contents in the peel (Fig. 9B). Degreening was also accompanied by a gradual decline in the peel contents of lutein, β-carotene, and α-carotene (Fig. 9C).

We then examined the expression patterns of selected genes induced by ethylene and/or low temperature as identified from the RNA-seq data. On-tree peel degreening coincided with up-regulation of *ClPPH* and down-regulation of *ClLHCB2* (Fig. 9D), both of which we had established to be influenced by low temperature (Figs 4B, 6B). However, the ethylene-specific *ClCLH1* did not show any significant changes in expression. On-tree degreening was also accompanied by up-regulation of all three of the carotenoid metabolism genes that we analysed, namely *ClPSY1*, *ClLCYb2a*, and *ClCHYb1* (Fig. 9E), which we had established to be up-regulated by low temperature (Fig. 5B). Among the genes encoding TFs, the ethylene-specific *ClERF114* did not show any significant expression changes, whereas both *ClERF3* and *ClbHLH25* were up-regulated from 30 October onwards, when the minimum temperatures were below 13 °C (Fig. 9F). Taken together, these results demonstrated strong similarities between on-tree and low temperature-modulated degreening, and further highlighted dissimilarities with ethylene-induced changes.

## Discussion

A close association between low temperature and peel colouration has been described in several citrus species (Carmona *et al.*, 2012b; Manera et al., 2012, 2013). In agreement with these findings, our results showed that moderately low storage temperatures (5–20 °C) promoted peel degreening in lemon fruit, and induced substantial changes in chlorophyll and carotenoid contents (Fig. 2). To date, the molecular mechanisms involved have not been determined, but because of the known involvement of ethylene in citrus degreening (Purvis and Barmore, 1981; Shemer *et al*., 2008; Yin *et al.*, 2016), it has been suggested that it might play a role in the process, as trace levels of physiologically active System I ethylene are thought to be bound in tissues (Goldschmidt *et al.*, 1993; Carmona *et al.*, 2012b). In this current study, we present conclusive data demonstrating that low temperature can modulate natural peel degreening in lemon fruit independently of the ethylene signal.

Ethylene-induced degreening is completely inhibited by pre-treatment with 1-MCP (Fig. 1; Jomori *et al.*, 2003; McCollum and Maul, 2007; Li *et al.*, 2016) and 1-MCP treatment has been shown to inhibit the ripening process in fruit that have a strong requirement for ethylene to ripen (Watkins, 2006). In higher plants, ethylene receptors act as negative regulators (Hua and Meyerowitz, 1998), and their binding by ethylene subjects them to degradation via the ubiquitin-proteasome pathway (Kevany *et al.*, 2007). 1-MCP is assumed to irreversibly bind to and phosphorylate the receptors (Kamiyoshihara *et al.*, 2012) with a higher affinity than ethylene (Jiang *et al.*, 1999), resulting in relatively stable complexes that suppress signalling even in the presence of ethylene. If endogenous ethylene is physiologically active during storage, then its action should be suppressed by the application of antagonists such as 1-MCP. In this study, however, it was surprising that peel degreening elicited by low temperature was not abolished by repeated 1-MCP treatments (Fig. 2), indicating that it most likely occurred in an ethylene-independent manner.

This conclusion was supported by the transcriptomic data that we obtained. Genes that were differentially regulated during lemon fruit peel degreening could be categorized into three distinct groups, first those regulated specifically by ethylene, second those regulated exclusively by low temperature, and third those regulated by ethylene or low temperature (Fig. 3). Ethylene-specific genes such as *ClCLH1* and *ClERF114* did not show any significant changes in expression during low temperature-modulated degreening (Figs 4B, 8B), indicating that ethylene signalling was non-functional in the stored fruit. In addition, our identification of low temperature-specific genes, including *ClCHYb1* and *ClERF3* (Figs 5B, 8C), strongly supports the concept that low temperature-modulated degreening involves a mechanism that operates outside the sphere of influence of ethylene. Although the third set of genes were differentially regulated by ethylene or low temperature, efforts to suppress the low-temperature regulation of key members (*ClPPH*, *ClLHCB2*, *ClPSY1*, *ClLCYb2a*, *ClNCED5*, and *ClbHLH25*) using repeated 1-MCP treatments were unsuccessful (Figs 4B, 5B, 6B, 7B, 8D). Thus, taken together with our physiological and metabolic data, it was evident that ethylene-independent regulation of peel degreening by low temperature was occurring in lemon fruit.

As chlorophyll degradation is the most obvious change leading to peel degreening (Fig. 1B, 2C), we examined the changes in expression of related genes that were induced by ethylene and low temperature. We found that ethylene treatment did indeed lead to increased expression levels of *ClCLH1* and *ClPPH* (Fig. 4B), in agreement with previous findings in various citrus fruit species (Jacob-Wilk *et al.*, 1999; Shemer *et al*., 2008; Yin *et al.*, 2016). During storage, however, there were minimal changes in the expression of *ClCLH1*, whereas *ClPPH* was up-regulated at all the tested temperatures from 5–20 °C, in agreement with the reduction in chlorophyll content. *ClPPH* encodes an enzyme with similar dephytilation activity as CLH (Schelbert *et al.*, 2009), thus providing a mechanism by which the loss of chlorophyll triggered by low temperature is regulated.

The carotenoid content of the peel decreased upon degreening in response to both ethylene and low temperature (Figs 1C, 2D). Such decreases are not uncommon as previous studies have also found similar results in lemon fruit during maturation, especially for lutein (Kato, 2012; Conesa *et al.*, 2019). Nevertheless, the yellowish appearance of degreened fruit (Figs 1A, 2A, B) could be attributed to the small but significant levels of lutein, β-carotene, and α-carotene that were present (Fig. 1C, 2D), the appearance of which might have been enhanced by the loss of chlorophyll. The observation that the expression of carotenoid metabolism genes such as *ClPSY1*, *ClLCYb2a*, and *ClCHYb1* were stimulated by ethylene or low temperature (Fig. 5) agreed with previous reports (Rodrigo and Zacarias, 2007; Matsumoto *et al.*, 2009); however, this is the first study to demonstrate that carotenoid metabolism is modulated by low temperature independently of ethylene.

The degradation of chlorophylls caused by exposure to ethylene or low temperature could also be facilitated by changes in photosystem proteins. The disruption of pigment-protein complexes is thought to be a crucial step in the chlorophyll degradation pathway (Barry, 2009). Thus, the stay-green gene *SGR*, which encodes a Mg dechelatase (Shimoda *et al.*, 2016), has been shown to aid the dis-aggregation of photosystem proteins, and in particular the light-harvesting chlorophyll *a*/*b*-binding (CAB) complex (Jiang *et al.*, 2011; Sakuraba *et al.*, 2012). Because photosystem proteins bind pigments, a large drop in their transcripts caused by ethylene or low temperature (Fig. 6) would possibly favour the accumulation of free chlorophylls that can easily be accessed by degradatory enzymes. Peng *et al.* (2013) have also reported that the transcript levels of *CitCAB1* and *CitCAB2* drastically decrease during ethylene-induced and natural peel degreening in ‘Ponkan’ mandarins. However, our results suggest that the decrease in the expression of photosystem-encoding genes during natural peel degreening could be stimulated by low temperature independently of ethylene.

Besides ethylene, various phytohormones including ABA, GA, and JA have been implicated in the colour changes that occur in the peel during the maturation of citrus fruit. Peel degreening has been shown to be accompanied by an increase in ABA content (Goldschmidt *et al.*, 1973), as well as in the expression of ABA biosynthetic and signalling elements (Kato *et al.*, 2006; Rodrigo *et al.*, 2006). In addition, exogenous ABA accelerates fruit ripening and enhances fruit colour development (Wang *et al.*, 2016), while ABA-deficient citrus mutants show a delay in the rate of peel degreening (Rodrigo *et al.*, 2003). In our study, ABA levels increased in fruit treated with ethylene and in fruit stored at low temperatures (Fig. 7C), and this was accompanied by an increase in the expression of ABA biosynthetic and signalling genes (Fig. 7A, B). These findings, together with previous reports, suggest that ABA has a positive regulatory role in both ethylene-induced and low temperature-modulated peel degreening in lemon fruit. GA, on the other hand, is known to retard colour changes in the peel, and this effect is seen when it is applied to green citrus fruit (Alós *et al.*, 2006; Rodrigo and Zacarias, 2007; Ríos *et al*., 2010). It was therefore logical that the transcript levels of the GA biosynthetic gene *ClGA20ox2* decreased, whereas those of the degradatory genes *ClGA2ox4* and *GA2ox8* increased during peel degreening caused by ethylene or low temperature (Fig. 7A). JAs have largely been studied to date in the context of plant adaptive responses to various biotic and abiotic stresses (Zhang *et al.*, 2020). However, they have also been shown to promote fruit ripening in citrus (Zhang *et al.*, 2014), strawberry (Concha *et al.*, 2013), and tomato (Liu *et al.*, 2012). This is consistent with our findings, as degreening in lemon fruit was accompanied by an increase in the levels of JA-Ile (Fig. 7C), which is the active conjugate of JA. In addition, the expression of a large number of JA biosynthetic and signalling-related genes were independently upregulated by ethylene treatment or low temperature (Fig. 7A).

Developmentally regulated plant processes such as peel degreening are typically influenced by TFs. Various TFs in Arabidopsis such as NACs (AtNAC046 and AtORE1), bHLHs (AtPIF4 and AtPIF5), and ethylene insensitive 3 (AtEIN3) have been shown to significantly enhance leaf senescence by promoting the activity of genes related to chlorophyll degradation (Song *et al.*, 2014; Qiu *et al.*, 2015; Zhang *et al.*, 2015; Oda-Yamamizo *et al.*, 2016). In broccoli (*Brassica oleracea*), the MYB, bHLH, and bZIP gene families are associated with chlorophyll metabolism while NACs and ERFs regulate carotenoid biosynthesis (Luo *et al.*, 2019). CitERF6 and CitERF13 have also been associated with chlorophyll degradation during ethylene-induced and natural peel degreening in citrus (Yin *et al.*, 2016; Li *et al.*, 2019). In our present work, the expression patterns of genes encoding a wide range of TF families suggested that the ethylene-induced and low temperature-modulated peel degreening pathways were distinct in lemon fruit (Fig. 8). Thus, ethylene-induced degreening is most likely to be regulated by ethylene-specific TFs such as *ClERF114* (Fig. 8B) and shared TFs such as *ClbHLH25* (Fig. 8D), whereas low temperature-modulated degreening could be regulated by specific TFs such as *ClERF3* (Fig. 8C), as well as shared ones such as *ClbHLH25* (Fig. 8D).

In our study, low temperature also appeared to play a prominent role in natural peel degreening during on-tree maturation of the lemon fruit. Peel degreening and the associated reduction in the content of chlorophylls and carotenoids coincided with the gradual decline in minimum ambient temperatures to below 13 °C (Fig. 9A–C). Previous studies have also demonstrated that peel degreening in most citrus fruit progresses as the ambient temperature decreases (Manera et al., 2012, 2013; Rodrigo *et al.*, 2013; Conesa *et al.*, 2019). It is intriguing that *ClCLH1* and *ClERF114*, which we identified as exhibiting an ethylene-specific pattern of expression (Figs 4B, 8B), did not show any significant changes in expression during on-tree degreening (Fig. 9D, F). Previous studies have also demonstrated that *ClCLH1* homologues in other citrus fruit exhibit a dramatic induction in response to ethylene treatment and yet lack a measurable increase in expression during natural degreening (Jacob-Wilk *et al.*, 1999; Yin *et al.*, 2016). We suggest that this discrepancy could be due to a lack of a functional ethylene signalling during on-tree maturation, given that *ClCLH1* was only up-regulated in the presence of ethylene (Fig. 4B). In contrast, genes that responded to low temperatures during storage, namely *ClPPH*, *ClLHCB2*, *ClPSY1*, *ClLCYb2a*, *ClCHYb1*, *ClERF3*, and *ClbHLH25*, also exhibited similar expression patterns during on-tree maturation (Fig. 9D–F), indicating that they were also involved in the on-tree peel degreening processes. These similarities between low temperature-induced and on-tree gene expression patterns, coupled with their dissimilarities to ethylene-induced changes, provide clear evidence to suggest that on-tree peel degreening responses are modulated by low temperature independently of ethylene in lemon fruit.

It is also important to note that many genes were differentially expressed in fruit at 5 °C and yet the rate of peel degreening was significantly slower than in fruit at 10–20 °C. This was probably due to low activity of the enzymes associated with degreening at 5 °C, as low temperature is known to generally decrease enzyme activity in plants (Jin *et al.*, 2009; Yun *et al.*, 2012).

The regulation of fruit ripening by low temperature is not unique to citrus fruit. Previous studies have also demonstrated its role, either independently or in concert with ethylene, in the regulation of fruit ripening in various species such as kiwifruit (Mworia *et al.*, 2012; Asiche et al., 2017, 2018; Mitalo et al., 2018*b*, 2019*a*, 2019*b*), pears (El-Sharkawy *et al.*, 2003; Mitalo *et al.*, 2019c), and apples (Tacken *et al.*, 2010). From an ecological perspective, the primary purpose of ripening is to make fruit attractive to seed-dispersing organisms. To ensure their future survival, most temperate fruit species are faced with the challenge of dispersing their seeds in time before the onset of harsh winter conditions. Therefore, temperature drops associated with autumn might provide an alternative stimulus for inducing ripening in fruits such as citrus that lack a functional ethylene signalling pathway during maturation.

**Fig. 10.**
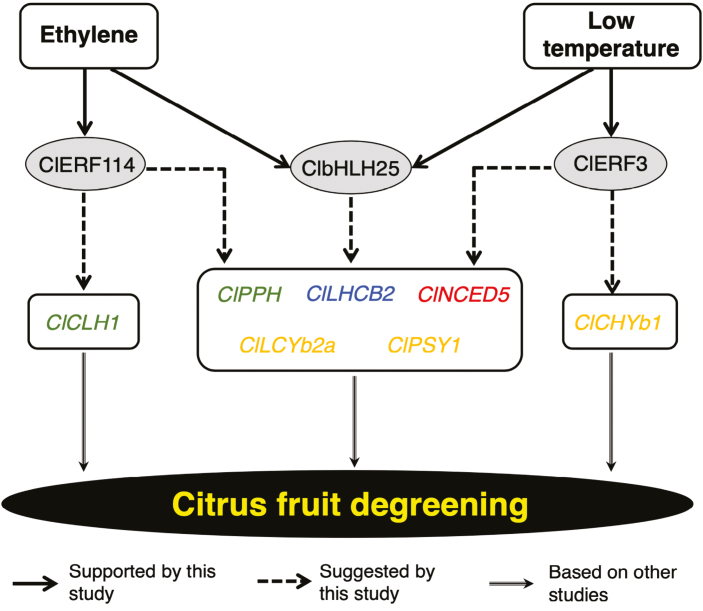
Proposed model for the distinct ethylene-dependent and low temperature-modulated peel degreening in lemon fruit. Uniquely ethylene-regulated transcription factors (TFs) are represented by ClERF114 while low temperature-specific TFs are represented by ClERF3. An additional set of TFs (represented by ClbHLH25) are shared between the ethylene and low-temperature regulatory pathways. Exposure to ethylene or low temperature induces changes in the levels of these unique and shared TFs, triggering the expression of various genes associated with chlorophyll degradation (green), carotenoid metabolism (orange), photosystem proteins (blues), and phytohormones (red).

Based on the results of this study, we present a simplified model that summarizes the distinct ethylene-dependent and low temperature-modulated regulatory mechanisms of peel degreening in citrus fruit (Fig. 10). According to this model, both ethylene and low temperature cause changes in the levels of unique TFs (such as the ethylene-specific ClERF114- and the low temperature-specific ClERF3) as well as in shared TFs such as ClbHLH25. These then trigger the expression of various genes associated with chlorophyll degradation, carotenoid metabolism, photosystem disassembly, and phytohormones, resulting in peel degreening. In future studies, we aim to identify the direct and indirect targets of the low temperature-regulated genes that we have identified here, and thus to determine the molecular bases for the modulation of peel degreening and fruit ripening in general.

## Supplementary data

Fig. S1. Chromatogram showing the carotenoids identified in the peel of lemon.

Fig. S2. Selected GO terms enriched among DEGs responding to ethylene, and storage at 5 °C and 15 °C.

Table S1. Liquid chromatography conditions for phytohormone analysis.

Table S2. Parameters used for LC-ESI-MS/MS analysis of phytohormones.

Table S3. Primer sequences used for RT-qPCR.

Table S4. DEGs exclusively responding to ethylene.

Table S5. DEGs responding to ethylene or to low temperature at 5 °C.

Table S6. DEGs responding to ethylene or to low temperature at 15 °C.

Table S7. DEGs responding to either ethylene, or to low temperatures at 5 °C or 15 °C.

Table S8. DEGs exclusively responding to low temperature at 5 °C.

Table S9. DEGs responding to low temperature at either 5 °C or 15 °C.

Table S10. DEGs exclusively responding to low temperature at 15 °C.

Table S11. Selected DEGs associated with peel degreening.

eraa206_suppl_Supplementary-Figures-S1-S2

eraa206_suppl_Supplementary-Tables-S1-S2

eraa206_suppl_Supplementary-Tables-S3-S11

## Data deposition

The RNA-seq data generated in this study have been deposited in the DDBJ Sequence Read Archive (accession numbers: DRR216064–DRR216079).
