# Supplementary material for: Low temperature modulates natural peel degreening in lemon fruit independently of endogenous ethylene
Source: J Exp Bot. 2020 May 6;71(16):4778–96. doi: 10.1093/jxb/eraa206 (PMC7410192; doi:10.1093/jxb/eraa206)
Supplement: eraa206_suppl_Supplementary-Figures-S1-S2 [file eraa206_suppl_supplementary-figures-s1-s2.pdf]

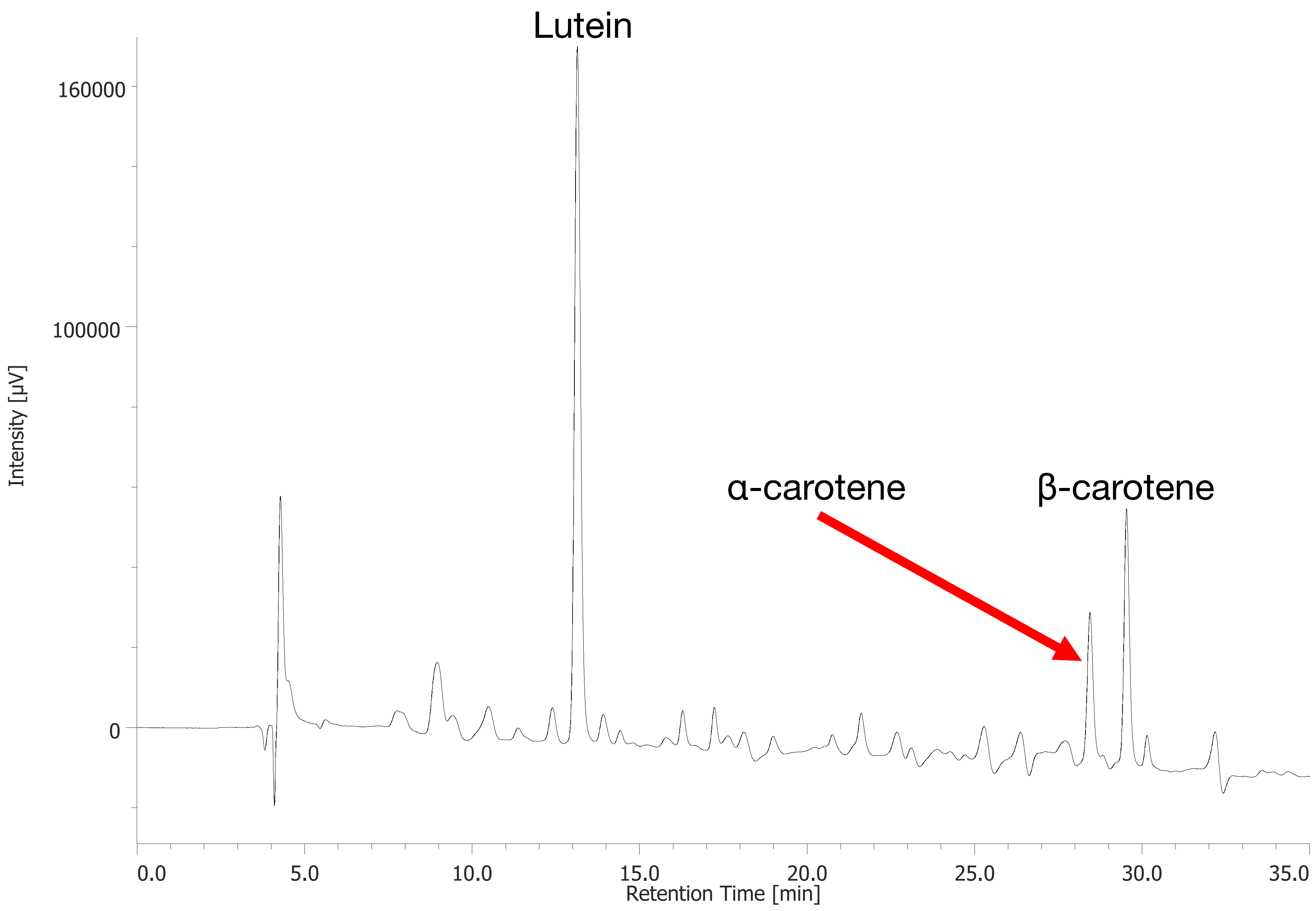

Fig. S1. Chromatogram showing the major carotenoid pigments identified in the flavedo of lemom fruit.

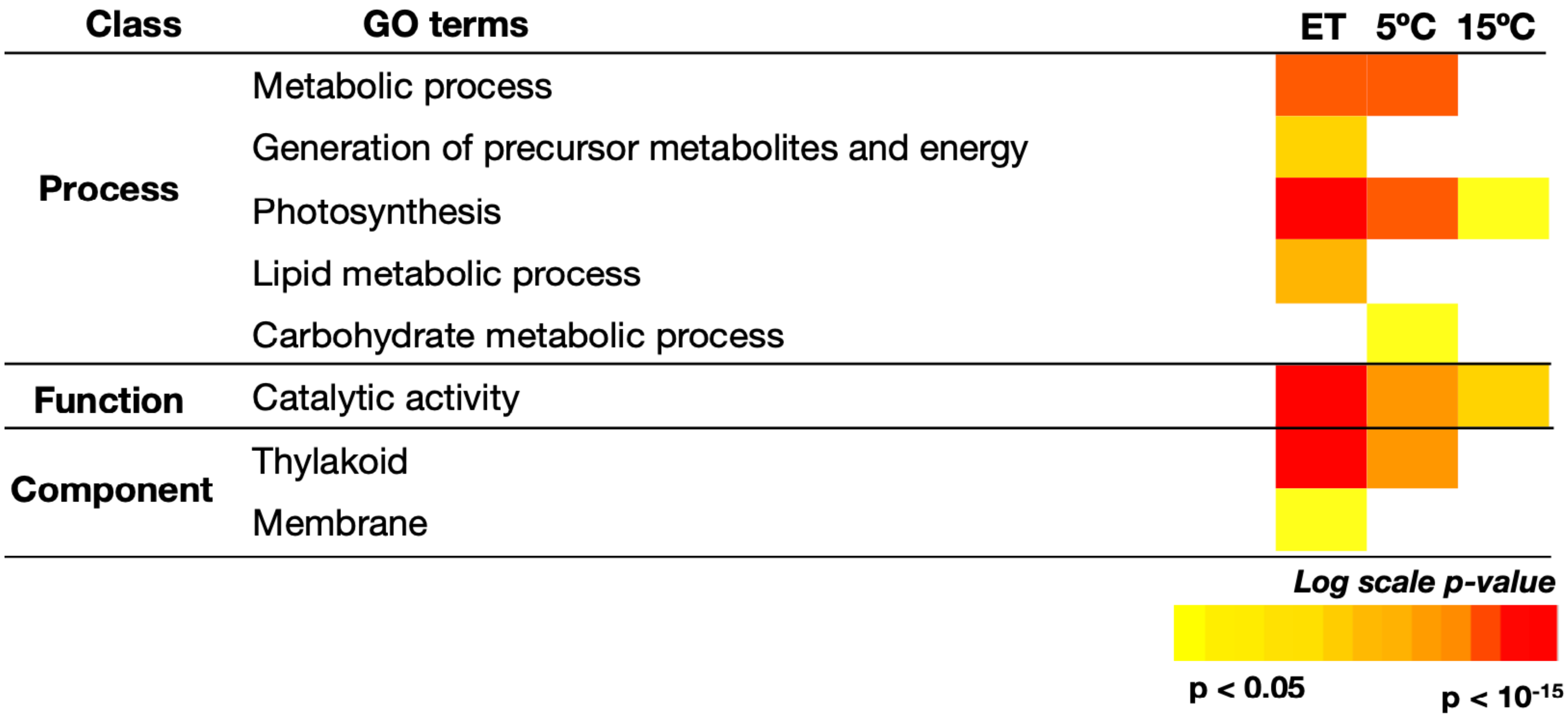

Fig. S2. Selected GO terms enriched among the DEGs identified in lemon fruit exposed to ethylene, 5ºC and 15ºC.
