# Supplementary material for: Low temperature modulates natural peel degreening in lemon fruit independently of endogenous ethylene
Source: J Exp Bot. 2020 May 6;71(16):4778–96. doi: 10.1093/jxb/eraa206 (PMC7410192; doi:10.1093/jxb/eraa206)
Supplement: eraa206_suppl_Supplementary-Tables-S1-S2 [file eraa206_suppl_supplementary-tables-s1-s2.pdf]

Supplementary Table 1. Liquid chromatography conditions. MeCN: methyl cyanide (acetonitrile), SA: salicylic acid.

Method No.	Hormone	Solvent A	Solvent B	Gradient (Composition of solvent B)
**1**	Acid	Water containing 0.01 % acetic acid	MeCN containing 0.05 % acetic acid	3 to 55 % 22 min
**2**	CK	Water containing 0.01 % acetic acid	MeOH containing 0.2 % acetic acid	3 to 97 % 16 min
**3**	SA	Water containing 0.1 % formic acid	MeCN containing 0.1 % formic acid	3 to 98 % 10 min

Supplementary Table 2. Parameters for LC-ESI-MS/MS analysis (Agilent 1260-6410). GA_1: gibberellin A_1, IAA: indole-3-acetic acid, ABA: abscisic acid, JA: jasmonic acid, GA_4: gibberellin A_4, JA-Ile: jasmonoyl-*L*-isoleucine, tZ: *trans*-zeatin, iP: N6-isopentenyladenine, SA: salicylic acid.

	LC method	Retention time on LC (min)	ESI	MS/MS transitions for quantifications (*m/z*)	Collision energy (V)	Fragmentor V
GA_1<br>D_2-GA_1	1	8.2	-	347/273<br>349/275	18	160
IAA<br>D_2-IAA<br>$^{13}C_6$-IAA<br>D_7-IAA	1	9.2	+	176/130<br>178/132<br>182/136<br>183/135, 136, 137	15	90
ABA<br>D_6-ABA	1	11.5	-	263/153<br>269/159	4	130
JA<br>D_2-JA<br>D_5-JA	1	13.0	-	209/59<br>211/59<br>214/62	11	135
GA_4<br>D_2-GA_4	1	15.4	-	331.2/257<br>333.2/259	20	160
JA-Ile<br>$^{13}C_6$-JA-Ile	1	16.2	-	321.2/130<br>338.4/136.2	17	140
tZ<br>D5-tZ	2	8.1	+	220.3/136.3<br>225.3/136.3, 137.3	12	100
iP<br>D_6-iP	2	12	+	204.4/136.4<br>210.4/137.4	11	110
SA<br>D_4-SA	3	5.3	-	137/93<br>141/97	15	90
